# GSK343, an Inhibitor of Enhancer of Zeste Homolog 2, Reduces Glioblastoma Progression through Inflammatory Process Modulation: Focus on Canonical and Non-Canonical NF-κB/IκBα Pathways

**DOI:** 10.3390/ijms232213915

**Published:** 2022-11-11

**Authors:** Sarah Adriana Scuderi, Alessia Filippone, Rossella Basilotta, Deborah Mannino, Giovanna Casili, Anna Paola Capra, Giulia Chisari, Lorenzo Colarossi, Serena Sava, Michela Campolo, Emanuela Esposito, Irene Paterniti

**Affiliations:** 1 Department of Chemical, Biological, Pharmaceutical and Environmental Sciences, University of Messina, Viale Ferdinando Stagno D’Alcontres, 98166 Messina, Italy; 2 Istituto Oncologico del Mediterraneo, Via Penninazzo 7, 95029 Viagrande, Italy

**Keywords:** glioblastoma, histone methyl transferase enhancer of zeste homolog 2, inflammation, apoptosis, immune response

## Abstract

Glioblastoma (GB) is a tumor of the central nervous system characterized by high proliferation and invasiveness. The standard treatment for GB includes radiotherapy and chemotherapy; however, new therapies are needed. Particular attention was given to the role of histone methyltransferase enhancer of zeste-homolog-2 (EZH2) in GB. Recently, several EZH2-inhibitors have been developed, particularly GSK343 is well-known to regulate apoptosis and autophagy processes; however, its abilities to modulate canonical/non-canonical NF-κB/IκBα pathways or an immune response in GB have not yet been investigated. Therefore, this study investigated for the first time the effect of GSK343 on canonical/non-canonical NF-κB/IκBα pathways and the immune response, by an in vitro, in vivo and ex vivo model of GB. In vitro results demonstrated that GSK343 treatments 1, 10 and 25 μM significantly reduced GB cell viability, showing the modulation of canonical/non-canonical NF-κB/IκBα pathway activation. In vivo GSK343 reduced subcutaneous tumor mass, regulating canonical/non-canonical NF-κB/IκBα pathway activation and the levels of reactive oxygen species (ROS), malondialdehyde (MDA), and superoxide dismutase (SOD). Ex vivo results confirmed the anti-proliferative effect of GSK343 and also demonstrated its ability to regulate immune response through CXCL9, CXCL10 and CXCL11 expression in GB. Thus, GSK343 could represent a therapeutic strategy to counteract GB progression, thanks to its ability to modulate canonical/non-canonical NF-κB/IκBα pathways and immune response.

## 1. Introduction

Glioblastoma (GB) is the most common and aggressive cancer of the central nervous system (CNS) with a global incidence of 10 cases per 100,000 people per year [1]. The vast majority of GB develops rapidly de novo in elderly patients, without clinical or histologic evidence of a less malignant precursor lesion [2]; whereas secondary GB progresses from low-grade diffuse astrocytoma or anaplastic astrocytoma [2]. Its histopathological features include cellular polymorphism, nuclear atypia, mitotic activity, vascular thrombosis, microvascular proliferation, and necrosis [3]. Current standard therapy for GB includes surgical resection, followed by radiotherapy and chemotherapy with temozolomide (TMZ), an oral alkylating agent [4]. Although the clinical treatment options are multiple, the survival rate for patients with GB remains very low and additional therapies are needed [1]. Studies have demonstrated that more than 140 gene mutations are involved in GB [5,6]; between these, EGFR (epidermal growth factor receptor), TP53 (tumor protein p53), PTEN (phosphatase and tensin homolog), PIK3CA (phosphoinositide-3-kinase catalytic alpha) resulted in being the most frequently altered genes in GB [6,7]. Although the exact molecular mechanisms of GB pathogenesis are not fully understood, studies revealed that epigenetic alterations significantly contribute to GB progression [8]. Particularly, various reports have focused on the link between the enhancer of zeste homolog 2 (EZH2) and cancer [9,10,11]. EZH2 is a histone H3 lysine methyltransferase encoded by the EZH2 gene, that participates in histone methylation and transcriptional repression [12]. EZH2 is the catalytic subunit of polycomb repressive complex 2 (PRC2) belonging to the epigenetic regulatory factor PcG proteins family which can alter downstream target genes expression by trimethylation of Lys-27 in histone 3 (H3K27me3) [13]. EZH2 is able to regulate several cell processes as the differentiation of hematopoietic stem and progenitor cells, immune cell development, including T, B, and natural killer cells, cell cycle progression, autophagy and apoptosis [9]; however, its overexpression or dysregulation may contribute to cancer initiation and progression [12]. Not much is known about the interaction of EZH2 with the NF-κB signaling pathway, the major regulator of inflammation, and immune response in GB. The activation of NF-κB involves two signaling pathways, the canonical and non-canonical pathways, both important for regulating immune and inflammatory responses [14]. The canonical NF-κB pathway responds to diverse stimuli, including ligands of various cytokine receptors, pattern-recognition receptors (PRRs), as well as the T-cell receptor (TCR) and B-cell receptor; it is mediated by the IKK complex, which consists of IKKα, IKKβ and the regulatory subunit IKK-γ/NF-κB essential modulator (NEMO) [14]. In contrast, the alternative or non-canonical pathway which plays a pivotal role in the cellular response to DNA damage, is tightly regulated by the NFκB-inducing kinase (NIK) [14]. In this regard, it has been demonstrated that in breast cancer cells, the EZH2 protein caused an activation or repression of the NF-κB pathway depending on the estrogen receptor (ER) status of the cancer cells [15]. In ER-negative cancer cells, EZH2 physically interacted with NF-κB heterodimers to promote the expression of a subset of NF-κB target genes; whereas in ER-positive cells, ER recruited EZH2-containing complexes to NF-κB target genes to epigenetically silence them via histone methylation [15]. A different interaction was found between NF-κB and EZH2 in endothelial cells infected with Kaposi sarcoma-associated herpesvirus in which latent viral genes were found to activate NF-κB, promoting inflammatory cascade activation and cancer progression [16]. De Donatis et al. [17] demonstrated a relationship between the non-canonical/canonical NF-κB pathways and EZH2 level in melanoma, suggesting that its inhibition could be a valid therapeutic strategy. Previous reports revealed that EZH2 acts as an oncogene in GB [18,19,20], promoting multiple glioma cellular processes, including cell cycle, cell invasion, and angiogenesis, which is thought to be responsible for cancer cells survival, drug resistance and tumor recurrence [19,21]. Furthermore, it has been demonstrated that EZH2 is involved in innate and adaptative immune response in cancer, promoting tumor development through a cell-extrinsic mechanism that involves the inhibition of the antitumor activity of NK cells [21,22]. The expression of EZH2 is directly related to GB malignancy grade and unfavorable survival; therefore, it could be recognized as a biomarker for diagnosis and prognosis of GB patients [20]. Given the evidence for EZH2 enzymatic gain-of-function being a cancer driver, the development of EZH2-specific inhibitors is an active and relevant area of investigation [10]. Since 2012, several potent and highly selective inhibitors of EZH2 methyltransferase activity have been developed including 3-deazaneplanocin A (DZNep), GSK126 and GSK343 [12,23]. Particularly attention was given to GSK343, a competitive inhibitor of methionine histone lysine methyltransferase EZH2 [24,25,26]; it has been reported that GSK343 powerfully inhibits EZH2 activity, suppressing the progression of various cancer types including ovarian cancer [27], osteosarcoma [28] neuroblastoma and glioma [26], promoting programmed cell death and autophagy processes [29]. Pharmacokinetic studies revealed that some EZH2 inhibitors such as GSK126 and MC3629 cross the BBB [30,31]; however, not much is known about the ability of GSK343 to traverse BBB, nevertheless, considering the similar molecular structure with GSK126, this could be a future objective to evaluate in the context of brain tumors [30]. However, although some of the biological properties of GSK343 in GB have been investigated over the past decade [25,32], many molecular interactions and cross-talks remain unknown such as the regulation of canonical and non-canonical NF-κB/IκBα pathways or immune response. Therefore, considering these assumptions, we aimed to evaluate for the first time the effect of GSK343 on canonical and non-canonical NF-κB/IκBα pathways and immune response by using in vitro, in vivo and ex vivo models of GB, so as to provide a new perspective on this complex clinical scenario.

## 2. Results

### 2.1. In Vitro Studies

#### 2.1.1. Effect of GSK343 Treatment on GB Cell Viability

**Figure 1:**
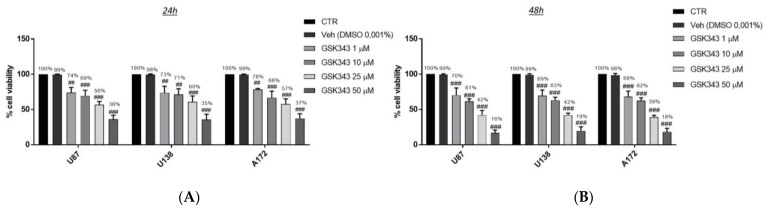
Effect of GSK343 treatment on human GB cell viability. MTT assay revealed that the treatment with GSK343 at the concentrations of 1, 10, 25 and 50 μM significantly reduced viability in U87, U138, and A172 cell lines, in a concentration-dependent manner both at 24 h (**A**) and 48 h (**B**) compared to the control group. Data are representative of at least three independent experiments. (**A**) ## *p* < 0.01 vs. Ctr; ### *p* < 0.001 vs. Ctr. (**B**) ### *p* < 0.001 vs. Ctr.

GB cell viability was assessed following 24 h and 48 h of treatment with GSK343 at increasing concentrations (1, 10, 25 and 50 μM). GSK343 treatment significantly reduced viability in all GB cell lines in the same way and in a concentration-dependent manner both at 24 h and 48 h compared to the control groups (Figure 1A,B). However, the treatment with GSK343 at 50 μM exerted an elevated toxicity in all three GB cell lines both at 24 h and 48 h, reducing viability by more than 40% and 20%, respectively, compared to the control groups. Furthermore, we decided to evaluate the cytotoxicity of GSK343 also in normal human astrocytes (NHA) cells as a control, showing that the treatment with GSK343 at the higher concentration of 50 μM decreased NHA viability both at 24 h and 48 h (73% and 69%, respectively) (Supplementary Figure S1A,B). Therefore, based on these results, we decided to continue analyzing GSK343 only at the concentrations of 1, 10 and 25 μM to reduce the level of toxicity. The IC_50_ values for the U87 cell line are 4.06 μM at 24 h and 4.68 μM at 48 h.

Since GSK343 showed similar effects on cell viability in all GB cell cultures, we decided to continue to analyze the effect of GSK343 only on the U87 cell line, because it represented one of the most frequently used cell lines in the field of GB [33].

#### 2.1.2. Effect of GSK343 Treatment on Canonical and Non-Canonical NF-κB/IκBα Pathways in U87 Cell Lysates

**Figure 2:**
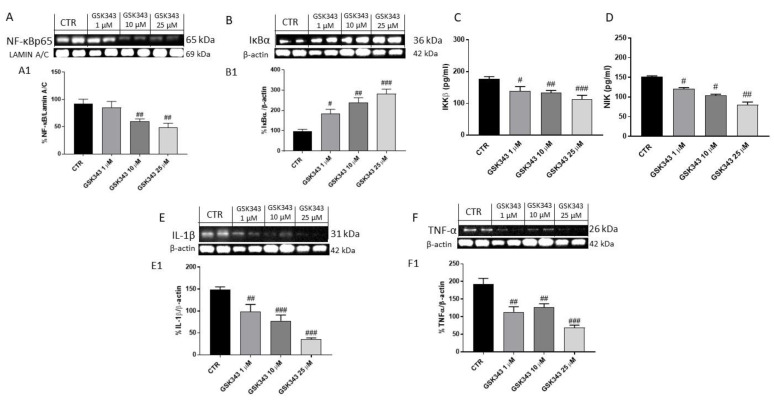
Effect of GSK343 treatment on NF-κB/IκBα pathway in U87 cell lysates. Our data demonstrated that the treatment with GSK343 at the concentrations of 1, 10 and 25 μM significantly reduced NF-κB (**A**), IKKβ expression (**C**) and restored IκBα expression (**B**). Moreover, GSK343 treatment at the concentrations of 1, 10 and 25 μM significantly reduced NIK level (**D**), and pro-inflammatory cytokines IL-1β (**E**) and TNFα expression (**F**). Data are representative of at least three independent experiments. (**A**) ## *p* < 0.01 vs. Ctr; (**B**) # *p* < 0.05 vs. Ctr; ## *p* < 0.01 vs. U87 cells; ### *p* < 0.001 vs. Ctr; (**C**) # *p* < 0.05 vs. U87 cells; ## *p* < 0.01 vs. U87 cells; ### *p* < 0.001 vs. U87 cells; (**D**) # *p* < 0.05 vs. Ctr; ## *p* < 0.01 vs. Ctr; (**E**) ## *p* < 0.01 vs. Ctr; ### *p* < 0.001 vs. Ctr; (**F**) ## *p* < 0.01 vs. Ctr; ### *p* < 0.001 vs. Ctr.

The effect of GSK343 on the NF-κB/IκBα pathway was evaluated in U87 cell lysates. Our results demonstrated that the treatment with GSK343 for 24 h at the concentrations of 10 and 25 μM was able to significantly reduce NF-κB expression and also at the lower concentration of 1 μM, restored IκB-α expression and decreased IKKβ in a concentration-dependent manner compared to the control group (Figure 2A–C). Additionally, we detected the effect of GSK343 on NF-κB-inducing kinase (NIK) level, a kinase that regulates the activation of the non-canonical NF-κB pathway, by ELISA kit, demonstrating that GSK343 treatment for 24 h was able to reduce its level (Figure 2D). Moreover, we decided to investigate the effect of GSK343 on pro-inflammatory cytokines expression such as IL-1β and TNF-α by Western blot analysis, showing that GSK343 treatment for 24 h at the concentrations of 1, 10 and 25 μM significantly decreased their expression compared to control group (Figure 2E,F).

#### 2.1.3. GSK343 Treatment Increased the Apoptotic Process in U87 Cell Lysates

**Figure 3:**
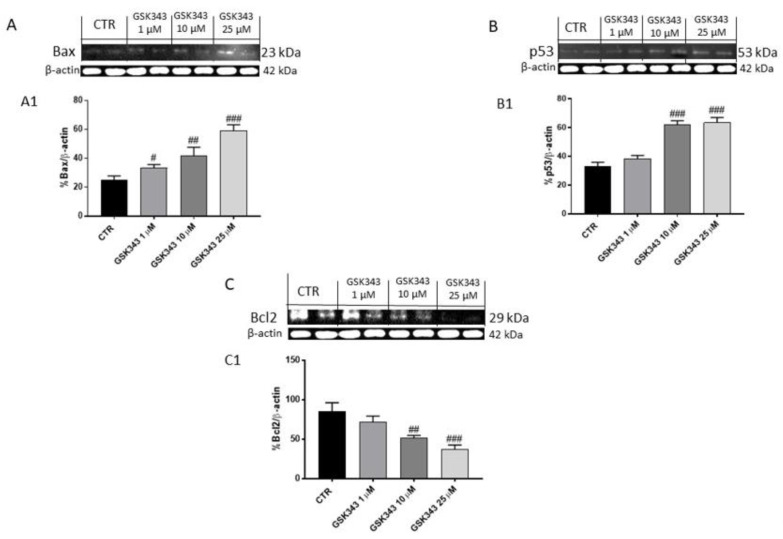
Effect of GSK343 treatment on apoptosis process in U87 cell lysates. Western blot analysis revealed that the treatment with GSK343 at the concentrations of 1, 10 and 25 μM significantly increased pro-apoptotic protein Bax and p53 expression, whereas Bcl2 expression was reduced following GSK343 treatment compared to the control group. Data are representative of at least three independent experiments. (**A**) # *p* < 0.05 vs. Ctr; ## *p* < 0.01 vs. Ctr; ### *p* < 0.001 vs. Ctr; (**B**) ### *p* < 0.001 vs. Ctr; (**C**) ## *p* < 0.01 vs. Ctr; ### *p* < 0.001 vs. Ctr.

The effect of GSK343 on the apoptotic pathway was investigated in U87 cell lysates. After 24 h of treatment, our data demonstrated that GSK343 at the concentrations of 1, 10 and 25 μM was able to increase the expression of pro-apoptotic protein as Bax, while p53 expression was notably increased only at the concentrations of 10 and 25 μM (Figure 3A,B); instead, anti-apoptotic Bcl2 protein expression was significantly reduced at 10 and 25 μM concentrations, as shown in Figure 3C.

#### 2.1.4. GSK343 Treatment Modulated Epithelial–Mesenchymal Transition and Metalloproteinases Expression in U87 Cell Lysates

**Figure 4:**
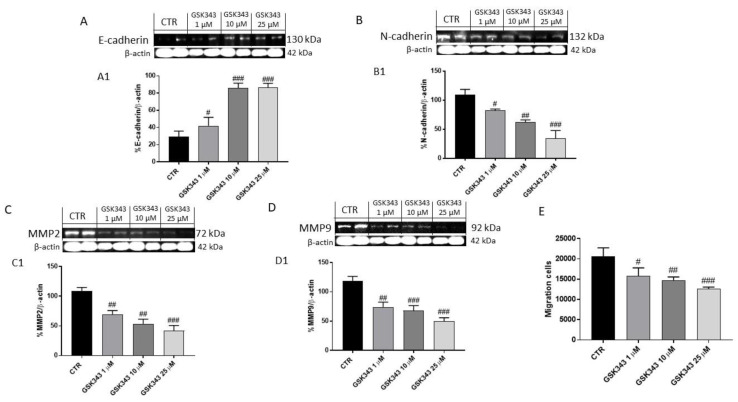
Effect of GSK343 treatment on E-cadherin, N-cadherin, MMP2 and MMP9 expression in U87 cell lysates. The treatment with GSK343 at the concentrations of 1, 10 and 25 μM increased E-cadherin and reduced N-cadherin expression compared to control group (**A**,**B**). Moreover, the treatment with GSK343 at the concentrations of 1, 10 and 25 μM significantly reduced MMP2, and MMP9 expression(**C**,**D**). The treatment with GSK343 decreased also U87 cells migration (**E**). Data are representative of at least three independent experiments. (**A**) # *p* < 0.05 vs. Ctr; ### *p* < 0.001 vs. Ctr; (**B**) # *p* < 0.05 vs. Ctr; ## *p* < 0.01 vs. Ctr; ### *p* < 0.001 vs. Ctr. (**C**) ## *p* < 0.01 vs. Ctr; ### *p* < 0.001 vs. Ctr; (**D**) ## *p* < 0.01 vs. Ctr; ### *p* < 0.001 vs. Ctr.(**E**) # *p* < 0.05 vs. Ctr; ## *p* < 0.01 vs. Ctr; ### *p* < 0.001 vs. Ctr.

Considering the involvement of EZH2 in epithelial–mesenchymal transition (EMT) [34], we decided to investigate the effect of GSK343 treatment on E-cadherin and N-cadherin protein levels by Western blot analysis. Our data demonstrated that the treatment with GSK343 for 24 h at the concentrations of 1, 10 and 25 μM was able to increase E-cadherin expression and reduce N-cadherin expression (Figure 4A,B).

Additionally, we investigated the effect of GSK343 treatment on matrix metalloproteinases (MMPs), particularly MMP2 and MMP9 expression, showing that the treatment with GSK343 for 24 h at the concentrations of 1, 10 and 25 μM was able to notably reduce their expression compared to the control group (Figure 4C,D). To confirm the effect of GSK343 on EMT and MMPs, we performed the cell migration assay, showing that the treatment with GSK343 significantly decreased U87 cell migration (Figure 4E).

### 2.2. In Vivo Studies

#### 2.2.1. Effect of GSK343 Treatment on Tumor Growth

**Figure 5:**
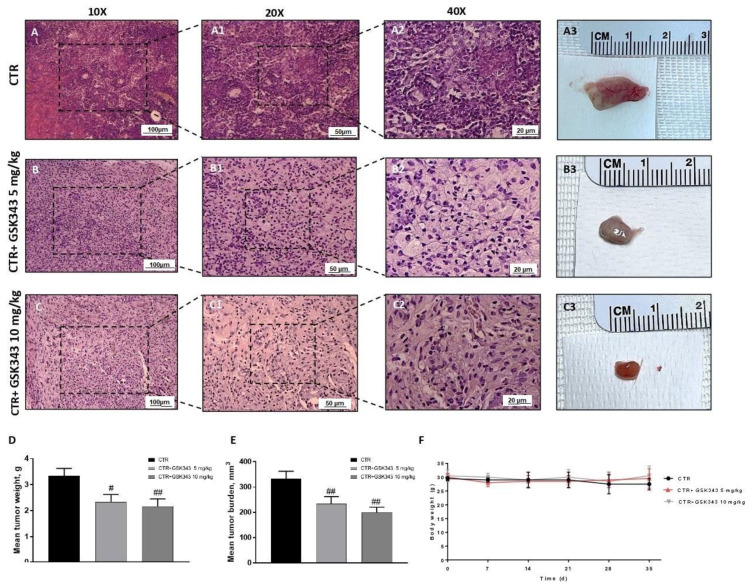
Effect of GSK343 treatment on tumor growth. Elevated nuclear pleomorphism was observed in the control group (**A**,**A1**,**A2**); however, GSK343 at doses of 5 mg/kg and 10 mg/kg significantly reduced it (**B**,**B1**,**B2**,**C**,**C1**,**C2**). The panels D and E showed a reduction in tumor weight and tumor burden, respectively, following GSK343 treatment at doses of 5 mg/kg and 10 mg/kg without encountering important weight differences (**F**). Sections were observed and photographed at 10×, 20× and 40× magnifications. Data are representative of at least three independent experiments. (**D**) # *p* < 0.05 vs. Ctr; ## *p* < 0.01 vs. Ctr; (**E**) ## *p* < 0.01 vs. Ctr.

The histological evaluation demonstrated that the control group was characterized by nuclear pleomorphism and high cellular density (Figure 5A,A1,A2). However, the treatment with GSK343, at doses of 5 mg/kg and 10 mg/kg, was able to significantly reduce it compared to the control group (Figure 5B1,B2,C1,C2). To better appreciate the histological evaluation, we showed the images in other 3 different areas, as demonstrated in the Supplementary Figure S2. Moreover, the treatment with GSK343 at doses of 5 mg/kg and 10 mg/kg significantly reduced the subcutaneous tumor mass (see macroscopic Figure 5A3,B3,C3), tumor weight as well as the tumor burden compared to the control group (Figure 5D,E). During the experiment, no important changes in animals’ weight were observed (Figure 5F).

#### 2.2.2. Effect of GSK343 Treatment on Canonical and Non-Canonical NF-κB/IκBα Pathways in U87-Xenograft Model

**Figure 6:**
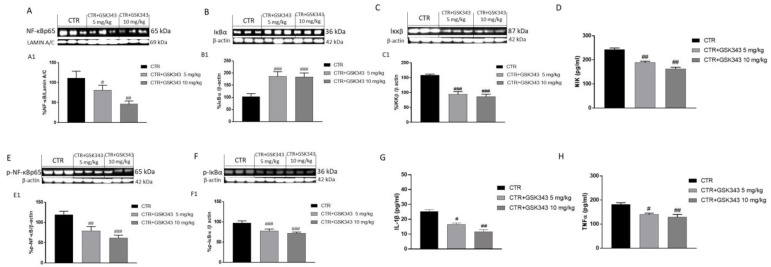
Effect of GSK343 on canonical and non-canonical NF-κB/IκBα pathways in the U87-xenograft model. The blots demonstrated that the treatments with GSK343 at doses of 5 mg/kg and 10 mg/kg significantly reduced NF-κB translocation into the nucleus (**A**), IKKβ expression (**C**) and restored IκBα cytosolic expression (**B**); NIK level was reduced following GSK343 treatment (**D**). Our results were confirmed for phosphorylated proteins p-NF-κB and p-IκB-α (**E**,**F**). Moreover, the ELISA assays demonstrated that GSK343 5 mg/kg and 10 mg/kg significantly reduced IL-1β (**G**) and TNF-α levels (**H**) compared to the control group. Data are representative of at least three independent experiments. (**A**) # *p* < 0.05 vs. Ctr; ## *p* < 0.01 vs. Ctr; (**B**) ### *p* < 0.001 vs. Ctr; (**C**) ### *p* < 0.001 vs. Ctr; (**D**) ## *p* < 0.01 vs. Ctr; (E) ## *p* < 0.01 vs. Ctr, ### *p* < 0.001 vs. Ctr; (**F**) ### *p* < 0.001 vs. Ctr; (**G**) # *p* < 0.05 vs, Ctr; ## *p* < 0.01 vs. Ctr; (**H**) # *p* < 0.05 vs. Ctr; ## *p* < 0.01 vs. Ctr.

Based on the in vitro results, we evaluated the effect of GSK343 treatment on canonical and non-canonical NF-κB/IκBα pathways also in the U87-xenograft model. Our results demonstrated that the control group was characterized by an increase in nuclear NF-κB expression and a decrease in cytosolic IκBα expression (Figure 6A,B). Moreover, the GB group demonstrated a significant increase in IKKβ expression (Figure 6C); however, the treatment with GSK343 at both doses was able to reduce NF-κB translocation into the nucleus and restore IκB-α cytosolic expression compared to the control group (Figure 6A,B), also reducing IKKβ expression (Figure 6C). Additionally, to prove that GSK343 was able to modulate non-canonical NF-κB/IκBα pathway activation, we also investigated NIK level by ELISA kit, demonstrating that the treatment with GSK343 at doses of 5 mg/kg and 10 mg/kg significantly decreased its level (Figure 6D). Our data on the NF-κB/IκBα pathway were also confirmed by evaluating phosphorylated proteins p-NF-κB and p-IκB-α by Western blot analysis (Figure 6E,F). Moreover, we decided to evaluate the effect of GSK343 treatment also on pro-inflammatory cytokines levels as IL-1β and TNF-α by ELISA kit, demonstrating that the control group was characterized by high levels of IL-1β and TNF-α; however, the treatment with GSK343 at doses of 5 mg/kg and 10 mg/kg significantly reduced their levels (Figure 6G,H).

#### 2.2.3. Effect of GSK343 Treatment on the Apoptotic Process in U87-Xenograft Model

**Figure 7:**
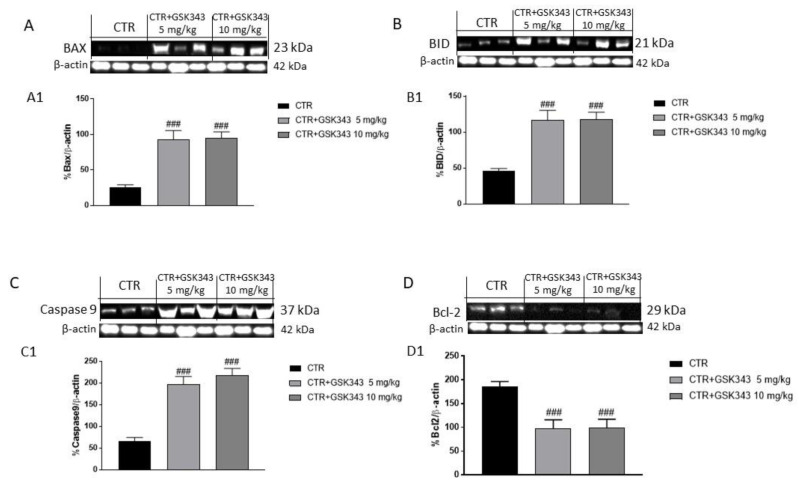
Effect of GSK343 on apoptosis process in the U87-xenograft model. The blots revealed that the treatments with GSK343 at doses of 5 mg/kg and 10 mg/kg significantly increased pro-apoptotic protein Bax, BID and caspase-9 expression (**A**–**C**), whereas Bcl2 expression was reduced following GSK343 treatment compared to control group (**D**). Data are representative of at least three independent experiments. (**A**) ### *p* < 0.001 vs. Ctr; (**B**) ### *p* < 0.001 vs. Ctr; (**C**) ### *p* < 0.001 vs. Ctr; (**D**) ### *p* < 0.001 vs. Ctr.

Apoptosis plays a key role in GB progression [35]. Therefore, to confirm the in vitro results, we decided to investigate the effect of GSK343 on apoptosis also in the U87-xenograft model by evaluating Bax, BID, caspase-9 and Bcl2 expression by Western blot analysis. Our results showed that the treatment with GSK343 at doses of 5 mg/kg and 10 mg/kg significantly increased pro-apoptotic Bax, BID and caspase-9 protein expression (Figure 7A–C), while Bcl2 expression was significantly reduced compared to control group (Figure 7D).

#### 2.2.4. Effect of GSK343 Treatment on Epithelial–Mesenchymal Transition and Metalloproteases Expression in U87-Xenograft Model

**Figure 8:**
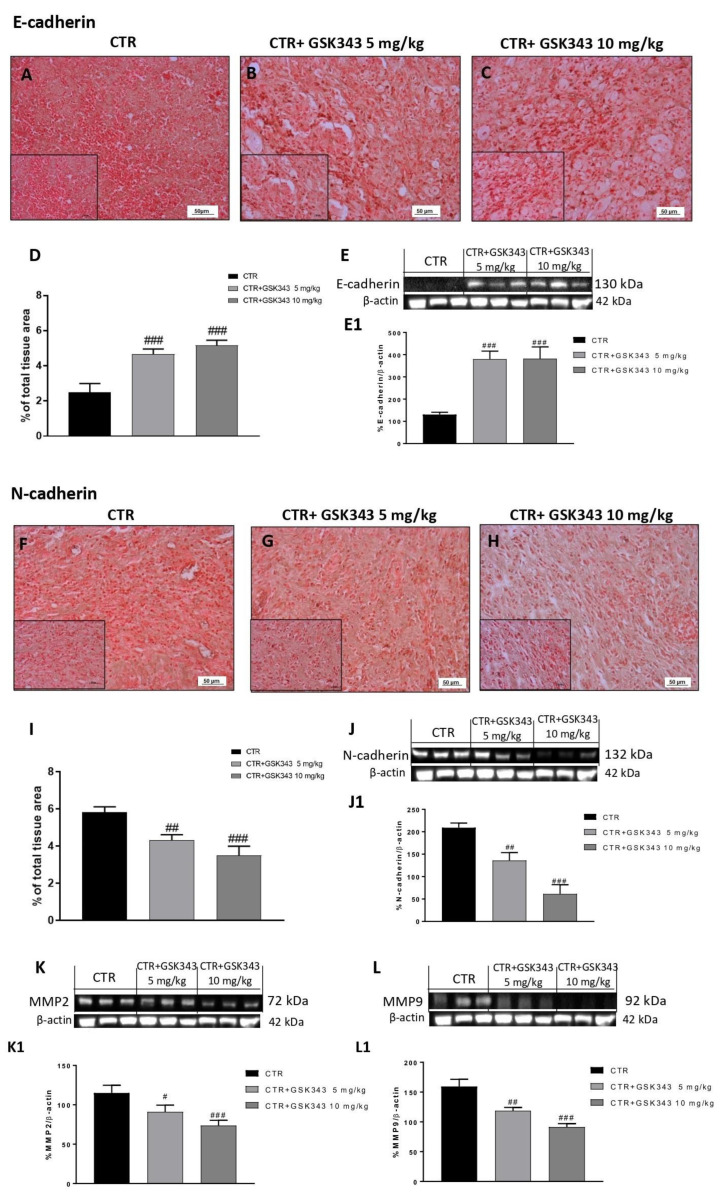
Effect of GSK343 on E-cadherin, N-cadherin, MMP2 and MMP9 expression in the U87-xenograft model. Immunohistochemical analysis revealed that GSK343 at doses of 5 mg/kg and 10 mg/kg significantly increased E-cadherin expression (**B**,**C**) compared to the control group (**A**). The data for E-cadherin was confirmed also by Western blot analysis (**E**). Furthermore, the treatments with GSK343 at doses of 5 mg/kg and 10 mg/kg (**G**,**H**) significantly reduced N-cadherin expression compared to the control group (**F**). The data for N-cadherin was also confirmed by Western blot analysis (**J**). The treatments with GSK343 at doses of 5 mg/kg and 10 mg/kg significantly reduced MMP2 and MMP9 expression compared to the control group (**K**,**L**). Sections were observed and photographed at 20x and 40x magnifications. (**D**) ### *p* < 0.001 vs. Ctr; (**E**) ### *p* < 0.001 vs. Ctr. (**I**) ## *p* < 0.01 vs. Ctr; ### *p* < 0.001 vs. Ctr; (**J**) ## *p* < 0.01 vs. Ctr; ### *p* < 0.001 vs. Ctr. (**K**) # *p* < 0.05 vs. Ctr; ### *p* < 0.001 vs. Ctr; (**L**) ## *p* < 0.01 vs. Ctr; ### *p* < 0.001 vs. Ctr.

Considering the in vitro results, we investigated the effect of GSK343 on the EMT process also in the U87-xenograft model by evaluating E-cadherin and N-cadherin expression. Immunohistochemical localization demonstrated that the control group was characterized by a decrease in E-cadherin expression and consequently an increase in N-cadherin expression (Figure 8A,F). However, the treatment with GSK343 at both doses was able to significantly increase E-cadherin and reduce N-cadherin expression (Figure 8B,C,G,H, respectively). The data for E-cadherin and N-cadherin were also confirmed by Western blot analysis, as shown in Figure 8E,J, respectively. Furthermore, we decided to evaluate metalloproteases (MMPs) levels also in the U87-xenograft model by Western blot analysis. Our data demonstrated that the treatment with GSK343 at doses of 5 mg/kg and 10 mg/kg significantly reduced MMP2 and MMP9 expression compared to the control group (Figure 8K,L), confirming the previous results.

#### 2.2.5. Effect of GSK343 on Oxidative Stress in U87-Xenograft Model

**Figure 9:**
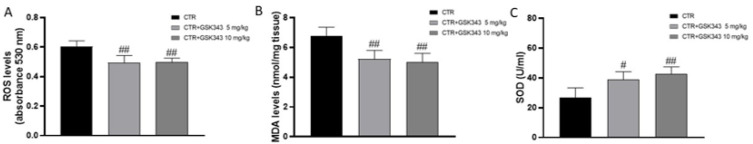
Effect of GSK343 on oxidative stress in U87-xenograft model. GSK343 5 mg/kg and 10 mg/kg decreased ROS, MDA, and increased SOD levels compared to the control group (**A**–**C**). Data are representative of at least three independent experiments. (**A**) ## *p* < 0.01 vs. Ctr; (**B**) ## *p* < 0.01 vs. Ctr; (**C**) # *p* < 0.05 vs. Ctr; ## *p* < 0.01 vs. Ctr.

Oxidative stress (OS) has been considered as one of many contributors in developing risk of cancer, including GB [36]; therefore, we decided to investigate the properties of GSK343 to modulate OS by evaluating the levels of reactive oxygen species (ROS) and malondialdehyde (MDA), a well-known marker of lipid peroxidation. Our data demonstrated that GSK343 at doses of 5 and 10 mg/kg was able to decrease ROS and MDA levels compared to the control group (Figure 9A,B). Moreover, we investigated the level of superoxide dismutase (SOD) enzyme, an endogenous antioxidant enzyme which protects cells against toxic levels of free radicals, showing that GSK343 at both doses increased its level, counteracting OS (Figure 9C).

#### 2.2.6. Effect of GSK343 Treatment on Primary GB Cell Viability

**Figure 10:**
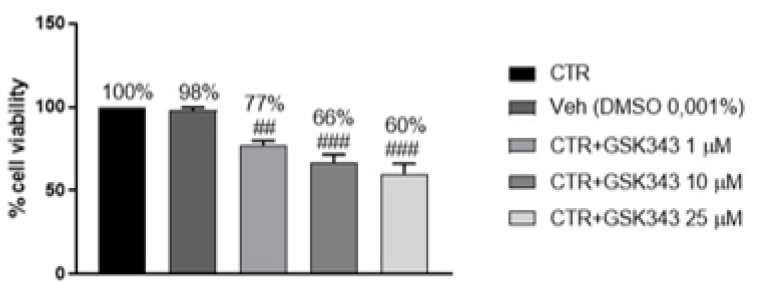
MTT assay revealed that GSK343 treatment (1, 10 and 25 μM) significantly reduced primary GB cell viability. Data are representative of at least three independent experiments. ## *p* < 0.01 vs. Ctr; ### *p* < 0.001 vs. Ctr.

GSK343 cytotoxicity was evaluated in primary GB cell culture obtained from patients, demonstrating that GSK343 treatment for 24 h at the concentrations of 1, 10 and 25 μM was able to significantly reduce primary GB cells viability compared to the control group, confirming its antiproliferative effect (Figure 10).

#### 2.2.7. Effect of GSK343 Treatment on Immune Response

**Figure 11:**
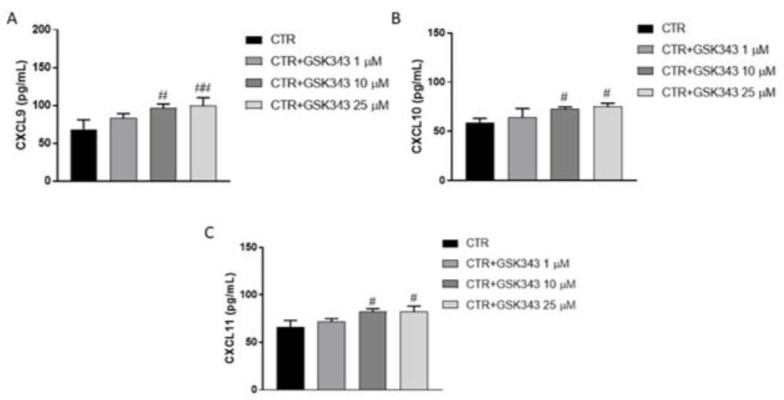
ELISA assay revealed that GSK343 treatment (10 and 25 μM) increased CXCL9, CXCL10 and CXCL11 levels (**A**–**C**) compared to the control group. No significance was revealed for the treatment 1 μM. Data are representative of at least three independent experiments. (**A**) # *p* < 0.05 vs. Ctr; ## *p* < 0.01 vs. Ctr; (**B**) # *p* < 0.05 vs. Ctr; (**C**) # *p* < 0.05 vs. Ctr.

Considering the role of EZH2 in immune response, we decided to evaluate the levels of chemokines CXCL9, CXCL10 and CXCL11, key mediators for the trafficking of antitumor immune cells, on primary GB cell culture by ELISA assay. Our data demonstrated that GSK343 treatment at the concentrations of 10 and 25 μM was able to increase CXCL9, CXCL10 and CXCL11 levels compared to the control group (Figure 11A–C).

## 3. Discussion

GB is the most common and aggressive malignant tumor of the central nervous system (CNS) [3]. In the last decade, many studies have focused on the role of EZH2 in GB [37,38], suggesting that its inhibition could represent a valid and alternative strategy to contrast cancer progression through apoptosis and autophagy modulation [32]. However, the relationship between EZH2 expression and canonical/non-canonical NF-κB pathway or immune response is not yet fully investigated in GB. Therefore, in this paper, we decided to evaluate for the first time the beneficial effect of GSK343, a highly potent and selective EZH2 inhibitor, in GB through canonical and non-canonical NF-κB pathway and immune response modulation, by an in vitro, in vivo and ex vivo model of GB. Firstly, we evaluated the cytotoxic effect of GSK343 at different concentrations in an in vitro model of GB using U87, U138 and A172 cell lines. Clearly, our data demonstrated that GSK343 treatment significantly reduced U87, U138 and A172 cell viability in a concentration-dependent manner both at 24 h and 48 h in the same way in all three cell cultures, highlighting its cytotoxic effect.

Various studies revealed that EZH2 is able to modulate many cellular processes, including inflammation and cell adhesion by targeting genes such as IL-1β and CDH13 [37]. Particularly, scientific evidence has focused on the crosstalk between EZH2 and NF-κB in cancer [39]. NF-κB is the major regulator of various cell processes, such as inflammation, proliferation, and apoptosis [14]. It has been demonstrated that aberrant activation of NF-κB could be associated with EZH2 overexpression or dysregulation in cancer [40]. Therefore, in this study we decided to investigate for the first time the effect of GSK343 on canonical and non-canonical NF-κB/IκBα pathways in GB. The activation of NF-κB involves two major signaling pathways, the canonical and non-canonical (or alternative) pathways, both being important for regulating immune and inflammatory responses despite their differences in signaling mechanism [14]. In the canonical NF-κB pathway, the activation of the IκB kinase (IKK/IKBK) complex (IKK) (composed of IKKα, IKKβ, and NF-κB essential modulator (NEMO)/IKKγ subunits) leads to IκBα phosphorylation by IKKβ [14]. In vitro, GSK343 treatment demonstrated the ability to reduce NF-κB and IKKβ expression, and consequently increase IκB-α expression in a concentration-dependent manner. Once activated, NF-κB induces the expression of various pro-inflammatory cytokines such as IL-1β and TNF-α which consequently promote the inflammatory process in GB [41]. In this context, our data revealed that the treatment with GSK343 at higher concentrations significantly reduced their levels compared to the control group, counteracting inflammation. On the other hand, we detected the capacity of GSK343 in GB to modulate the non-canonical NF-κB signaling pathway which is regulated by NF-κB-inducing kinase (NIK), demonstrating for the first time that in vitro GSK343 was able to significantly reduce NIK level and consequently the non-canonical NF-κB pathway activation. Additionally, in an in vivo study, we confirmed the ability of GSK343 to modulate canonical and non-canonical NF-κB/IκBα pathway activation, also evaluating the expression of phosphorylated proteins p-NF-κB and p-IκB-α. Despite the biological pathways underlying GB malignancy are still unclear, scientific evidence revealed the interconnection between the NF-κB/IκBα pathway and proto-oncogene non-receptor tyrosine kinase SRC [42,43]. SRC, a non-receptor tyrosine kinase protein that in humans is encoded by the SRC gene, drives GB invasion and progression through various processes modulation, such as epithelial-to-mesenchymal transition, angiogenesis, phosphorylation of transcription factors NF-κB and OS, which consequently promote tumor growth [42,43]. NF-κB plays a key role in cancer-related oxidative stress (OS), including GB [44]. Brain tumorigenesis has been associated with OS that is reflected by an imbalance between free radicals’ production and antioxidant mechanism which in turn induces damage to protein, lipid and deoxyribonucleic acid (DNA), promoting genomic instability [44]. Reactive oxygen species (ROS), mainly superoxide anion radical (O_2_−), hydroxyl radical (·OH), and hydrogen peroxide (H_2_O_2_) are involved in carcinogenesis, particularly in the tumor initiation and promotion phases [45]. Despite OS having a controversial role in GB [46,47], studies indicate that antioxidant therapy may be a means of treating tumors [44,47]. In this context, scientific evidence demonstrated that EZH2 overexpression has been linked with an increase of OS [47,48,49]. Indeed, we found an increase of ROS and MDA levels in GB, probably correlated to NF-κB/IκBα and EZH2 over-expression, and a decrease of antioxidant enzyme SOD level. However, GSK343 treatment was able to decrease ROS and MDA level and increase SOD enzyme level, reducing OS. Moreover, it has been demonstrated that EZH2 overexpression could suppress apoptosis in a variety of cancers, promoting cancer cell survival [32]. In this context, studies revealed that the downregulation of EZH2 expression in cancer is associated with an increase of apoptosis and a cell cycle arrest in the G0/G phases [34]. Therefore, considering the key role of apoptosis in GB pathogenesis [18], we decided to evaluate the effect of GSK343 on the apoptotic pathway in GB cells. In this context, our results demonstrated that GSK343 treatment significantly increased the expression of pro-apoptotic proteins such as Bax and p53, whereas Bcl2 expression was significantly reduced. Moreover, data on programmed cell death obtained in the in vitro model were confirmed by the in vivo xenograft model, through a significant increase in pro-apoptotic Bax, BID and caspase-9 protein expression, and a decrease in anti-apoptotic Bcl2 expression following GSK343 treatment. Studies demonstrated that overexpression of EZH2 in cancer is associated with epithelial-to-mesenchymal transition (EMT) [24,37]. EMT has been shown to be crucial in tumorigenesis enhancing metastasis, chemoresistance and tumor stemness [50]. It is characterized by the loss of epithelial characteristics and the gain of mesenchymal attributes in epithelial cells; this process is regulated by a complex network of signaling pathways and transcription factors [50]. Due to its emerging role as a pivotal driver of tumorigenesis, targeting EMT is of great therapeutic interest in counteracting metastasis and chemoresistance in cancer patients [50,51]. Our results demonstrated that GSK343 treatment in a concentration-dependent manner was able to reduce N-cadherin and increase E-cadherin expression. The in vitro results on EMT were also confirmed through the in vivo xenograft model, in which we proved that GSK343 treatment, in a dose-dependent manner, significantly restored E-cadherin expression and reduced N-cadherin expression. Scientific evidence revealed that several proteases as MMP2 and MMP9 are involved in many steps of cancer, including primary tumor growth, angiogenesis, invasion of the basement membrane and stroma, and metastatic process [52,53]. The expression of MMP2 and MMP9 in GB found that the treatment with GSK343 was able to significantly reduce their expression both in vitro and in vivo, counteracting cancer cell migration. Although GSK343 demonstrated numerous beneficial effects, including the ability to reduce subcutaneous tumor mass in vivo, we decided to perform an ex vivo model on primary GB cells obtained from patients to confirm the effects of GSK343. According to previous results, GSK343 exerted a cytotoxic effect also on primary GB cells, confirming its antiproliferative effect. Despite the modulatory effects of GSK343 on EMT, apoptosis and autophagy are well-known examples [26,32], not enough was identified about the ability of GSK343 to act on the immune system in GB environment. The host immune response to cancer cells is a potent mechanism for tumor suppression. In this regard, previous studies exploring the mechanisms of EZH2-mediated oncogenesis have largely focused on the cell-intrinsic mechanisms by which EZH2 regulates the expression of genes that are necessary for cancer cell proliferation and survival, promoting tumor development and progression [22]. It has been demonstrated that EZH2 promotes tumor development through a cell-extrinsic mechanism involving inhibition of the antitumor activity of NK cells, the major components of the innate immune response, which are generally recruited to tumor sites with chemokines, such as CXCL9, CXCL10 and CXCL11, to explicate their immune function [22,54]. In this context, for the first time, we found that GSK343 treatment was able to modulate the innate immune response, increasing CXCL9, CXCL10 and CXCL11 levels in GB, as a result of EZH2 inhibition, enhancing NK cell migration to tumor sites and consequently NK cell-mediated tumor growth inhibition. Therefore, the obtained results demonstrated the beneficial effect of GSK343, proposing that it could be an alternative therapeutic strategy to counteract GB progression thanks to its ability to modulate canonical and non-canonical NF-κB/IκBα pathways and immune response in GB.

## 4. Material and Methods

### 4.1. In Vitro Studies

#### 4.1.1. Cell Lines

Human GB cell lines: U87 MG (U87 MG ATCC^®^ HTB-14™ Homo sapiens brain likely glioblastomas), U138MG (U138 MG ATCC^®^ HTB-16™ Homo sapiens brain glioblastoma IV grade), and A172 (A172\ATCC^®^ CRL-1620™ Homo sapiens brain glioblastoma) were obtained from ATCC (American Type Culture Collection, Rockville, MD, USA). The GB human cell lines U87, U138 and A172 were cultured in 75 cm^2^ flask with, respectively, ATCC-formulated Eagle’s Minimum Essential Medium (Catalog No. 30-2003; ATCC, Rockville, MD, USA) for U138 and Dulbecco’s modified Eagle’s medium (DMEM—Sigma-Aldrich^®^ Catalog No. D5030; St. Louis, MO, USA) for U87 and A-172, both supplemented with antibiotics (Penicillin 1000 units—Streptomycin 0.1 mg/L, Sigma-Aldrich^®^ Catalog No. P4333; St. Louis, MO, USA), L-glutamine (GlutaMAX™, ThermoFisher Scientific^®^ Catalog No. 35050061; Waltham, MA, USA) and 10% (*v*/*v*) Fetal bovine serum (FBS, Sigma-Aldrich^®^ Catalog No. 12103C St. Louis, MO, USA). Normal human astrocytes (NHA) (Lonza, Walkersville, MD, USA) were used as normal counterparts [55,56]; NHA cells were grown in astrocyte medium-containing supplements (Allcells) with 10% FBS. All cell lines are maintained in the incubator with a humidified atmosphere containing 5% CO_2_ at 37 °C.

#### 4.1.2. Cell Treatment

NHA, U87, U138 and A172 cells were cultured at a density of 4 × 10^4^ cells/well in 96-well plates. After 24 h, cells were treated with GSK343 (Sigma-Aldrich^®^ Catalog No. 1346704-33-3; St. Louis, MO, USA) at increasing concentrations of 1 μM, 10 μM, 25 μM and 50 μM dissolved in culture medium with 0.001% of DMSO for 24 h and 48 h.

##### Experimental Groups

Control groups: U87, U138 and A172 cell lines were treated with culture medium.
Vehicle groups: U87, U138 and A172 cell lines were treated with 0.001% of DMSO dissolved in culture medium.
GSK343 1 μM group: U87, U138 and A172 cell lines were treated with GSK343 1μM dissolved in culture medium with 0.001% of DMSO.
GSK343 10 μM group: U87, U138 and A172 cell lines were treated with GSK343 10 μM dissolved in culture medium with 0.001% of DMSO.
GSK343 25 μM group: U87, U138 and A172 cell lines were treated with GSK343 25 μM dissolved in culture medium with 0.001% of DMSO.
GSK343 50 μM group: U87, U138 and A172 cell lines were treated with GSK343 50 μM dissolved in culture medium with 0.001% of DMSO.

Furthermore, we evaluated the cytotoxicity of GSK343 at the concentrations of 1, 10, 25 and 50 μM in NHA cells as a control for 24 h and 48 h (Supplementary Figure S1).

#### 4.1.3. MTT Assay

Cell viability was measured using a mitochondria-dependent dye for live cells (tetrazolium dye; MTT), as previously described by Campolo et al. [57]. Cells were pre-treated with increasing concentrations of GSK343 (1 μM, 10 μM, 25 μM and 50 μM) dissolved in a culture medium with 0.001% of DMSO for 24 h and 48h to determine high concentrations with high toxicity on cell viability. After 24h and 48h, cells were incubated at 37 °C with MTT (0.2 mg/mL) for 1 h. The medium was removed by aspiration and the cells were lysed with DMSO (100 μL). The extent of reduction of MTT to formazan was quantified by measurement of optical density at 550 nm (OD550) with a microplate reader. The half maximal inhibitory concentration (IC_50_) of GSK343 from the percentage viability of U87 cells was calculated using Graph Pad prism 7.04 software by the interpolation of the values in dose–response curves.

#### 4.1.4. Western Blot Analysis

Western blot analysis in U87 cell lysates was performed as previously described [58]. The following primary antibodies were used: anti-nuclear factor of kappa light chain-enhancer in B-cells (NF-κB) (1:500; Santa Cruz Biotechnology, Dallas, TX, USA; sc-8008), anti-inhibitor nuclear factor of kappa light chain-enhancer in B-cells alpha (IκBα) (1:500; Santa Cruz Biotechnology, Dallas, TX, USA; sc-1643); anti-E-cadherin (1:500; Santa Cruz Biotechnology, Dallas, TX, USA, sc-8426), anti-N-cadherin (1:500; Santa Cruz Biotechnology, Dallas, TX, USA, sc- 59987), anti-interleukin1β (IL-1β) (1:250 Santa Cruz Biotechnology, Dallas, TX, USA, sc-1251), anti-tumor necrosis factor-α (TNFα) (1:250; Santa Cruz Biotechnology, Dallas, TX, USA; sc-12744); anti-Bax (1:500; Santa Cruz Biotechnology, Dallas, TX, USA; sc-7480); anti-Bcl2 (1:500; Santa Cruz Biotechnology, Dallas, TX, USA; sc-7382); anti-p53 (1:500; Santa Cruz Biotechnology, Dallas, TX, USA; sc-126); anti-MMP2(1:500; Santa Cruz Biotechnology, Dallas, TX, USA sc-13595); anti-MMP9 (1:500; Santa Cruz Biotechnology, Dallas, TX, USA sc-13520). Antibody dilutions were made in PBS/5% *w*/*v* nonfat dried milk/0.1% Tween-20 (PMT) and membranes incubated overnight at 4 °C. Membranes were then incubated with secondary antibody (1:2000, Jackson ImmunoResearch, West Grove, PA, USA) for 1 h at room temperature. To ascertain the blots, they were loaded with equal amounts of protein lysate, they were also incubated with anti-βactin for cytosolic fraction (1:500; Santa Cruz Biotechnology; Dallas, TX, USA. sc-8432) and anti-lamin A/C for nuclear fraction (1:500; Santa Cruz Biotechnology; Dallas, TX, USA, sc-376248). Signals were detected with enhanced chemiluminescence (ECL) detection system reagent according to the manufacturer’s instructions (Thermo Fisher, Waltham, MA, USA). The relative expression of the protein bands was quantified by densitometry with BIORAD ChemiDocTMXRS + software. The densitometric values are normalized with Lamin A/C and β-actin and expressed as % of the control.

#### 4.1.5. Enzyme-Linked Immunosorbent Assay (ELISA) for NF-κB-Inducing Kinase (NIK) and IKKβ

The levels of NIK and IKKβ were measured by ELISA kit according to the manufacturer’s instructions in U87 cell culture supernatant (NIK ELISA kit Catalogue No: E024745; Biobool; IKKβ ELISA kit Catalogue No: ELH-IKKB-1; RayBiotech).

#### 4.1.6. Cell Migration Assay

Cell migration in U87 cells was evaluated using Cell Migration Assay Kit (Abcam, ab235693) according to the manufacturer’s instructions.

#### 4.1.7. Statistical Analysis

All values are expressed as mean ± standard error of the mean (SEM) of “n” observations. Each analysis was performed three times with three samples replicates for each one. The results were analyzed by one-way analysis of variance (ANOVA) followed by a Bonferroni post hoc test for multiple comparisons. A *p*-value of less than 0.05 was considered significant.

### 4.2. In Vivo Studies

#### 4.2.1. Cell Line

The human GB cell line U87 (U87MG ATCC^®^ HTB-14™ Homo sapiens brain Likely glioblastomas) was obtained from ATCC (American Type Culture Collection, Rockville, MD, USA). U87 cells were cultured in 75 cm^2^ flask with Dulbecco’s modified Eagle’s medium (DMEM—Sigma-Aldrich^®^ Catalog No. D5030; St. Louis, MO, USA) supplemented with antibiotics (Penicillin 1000 units—Streptomycin 0.1 mg/L, Sigma-Aldrich^®^ Catalog No. P4333; St. Louis, MO, USA), L-glutamine (GlutaMAX™, ThermoFisher Scientific^®^ Catalog No. 35050061; Waltham, Massachusetts, USA) and 10% (*v*/*v*) Fetal bovine serum (FBS, Sigma-Aldrich^®^ Catalog No. 12103C St. Louis, MO, USA) in a humidified atmosphere containing 5% CO_2_ at 37 °C.

#### 4.2.2. Animals

BALB/c nude female mice were obtained from Jackson Laboratory (Bar Harbor, Hancock, ME, USA) and housed in microisolator cages under pathogen-free conditions on a 12 h light/12 h dark schedule for a week. Animals were fed a standard diet and water ad libitum. Animal experiments followed Italian regulations on protection of animals used for experimental and other scientific purposes (DM 116192) as well as EU regulations (European Directive 2010/63/EU amended by Regulation 2019/1010).

#### 4.2.3. Experimental Design

Xenograft tumor model was performed, as previously described [59]. Mice were inoculated subcutaneously with 3 × 10^6^ human U87 cells in 0.2 mL of Phosphate-Buffered Saline (PBS) and 0.1 mL Matrigel (BD Bioscience, Bedford, MA). Once tumors were palpable (100 mm^3^), the animals were randomly divided into groups to receive vehicle or GSK343 treatment. GSK343 was administered at doses of 5 mg/kg and 10 mg/kg once daily via intraperitoneal (ip) injection every day for 21 days, as described by Bownes et al. [29]. The animals were humanely euthanized after 21 days of treatment and the subcutaneous tumors were excised and processed for several analysis such as histological evaluation and Western blot analysis. Tumor volumes were measured non-invasively by using an electronic caliper. The tumor burden was calculated using the following formula: 0.5 × length × width. The tumor volume was calculated using an empirical formula, V = 1/2 × ((the shortest diameter)2 × (the longest diameter)). The experiments were performed three times to verify the data.

##### Experimental Groups

Animals were randomly divided into 3 groups, as described below:

1. Control group (vehicle): intraperitoneal (ip) administration of PBS with 0.001% of DMSO.
2. Control group+ GSK343 5 mg/kg: intraperitoneal (ip) administration of GSK343 5 mg/kg dissolved in PBS with 0.001% of DMSO.
3. Control group+ GSK343 10 mg/kg: intraperitoneal (ip) administration of GSK343 10 mg/kg dissolved in PBS with 0.001% of DMSO.

#### 4.2.4. Histological Evaluation

Histological evaluation was performed, as previously described [60]. Tumor samples were fixed with 10% neutral formalin, embedded in paraffin, and sectioned at 7 µm. Sections were deparaffinized with xylene and then stained with hematoxylin and eosin. The slides were analyzed by a pathologist blinded to the treatment groups. All sections were analyzed using an Axiovision microscope (Zeiss, Milan, Italy). The images were shown at magnifications of 10× (100 μm of the bar scale) 20× (50 μm of the bar scale) and 40× (20 μm of the bar scale).

#### 4.2.5. Western Blot Analysis

Tumor samples from each mouse were suspended in extraction Buffer A (0.2 mM PMSF, 0.15 mM pepstatin A, 20 mM leupeptin, 1mM sodium orthovanadate), homogenized at the highest setting for 2 min, and centrifuged at 12,000 rpm for 4 min at 4 °C, as previously described [58]. Supernatants are the cytosolic fraction, whereas the pellets, containing enriched nuclei, were resuspended in Buffer B (1% Triton X-100, 150 mM NaCl, 10 mM Tris–HCl pH 7.4, 1 mM EGTA, 1mM EDTA, 0.2 mM PMSF, 20 mm leupeptin, 0.2 mM sodium orthovanadate) and centrifuged at 12,000 rpm for 10 min at 4 °C; supernatants are the nuclear fraction. Protein concentration was estimated by the Bio-Rad protein assay using bovine serum albumin as standard. Then, tumor samples, in equal amounts of protein, were separated on 12% SDS-PAGE gel and transferred to nitrocellulose membrane, as previously described. The following primary antibodies were used: anti-E-cadherin(1:500; Santa Cruz Biotechnology, Dallas, TX, USA; sc-8426); anti-N-cadherin(1:500; Santa Cruz Biotechnology, Dallas, TX, USA sc-59987); anti-MMP2(1:500; Santa Cruz Biotechnology, Dallas, TX, USA sc- 13595); anti-MMP9 (1:500; Santa Cruz Biotechnology, Dallas, TX, USA sc-13520); anti-Bax (1:500; Santa Cruz Biotechnology, Dallas, TX, USA; sc-7480); anti-Bcl2 (1:500; Santa Cruz Biotechnology, Dallas, TX, USA; sc-7382), anti-BID (1:500; Santa Cruz Biotechnology, Dallas, TX, USA; sc- 373939), anti-caspase9 (1:500, Santa Cruz Biotechnology, Dallas, TX, USA; sc- 56076); anti-nuclear factor of kappa light chain-enhancer in B-cells (NF-κB) (1:500; Santa Cruz Biotechnology, Dallas, TX, USA; sc-8008), anti-inhibitor nuclear factor of kappa light chain-enhancer in B-cells alpha (IκBα) (1:500; Santa Cruz Biotechnology, Dallas, TX, USA; sc-1643); anti-p-IκBα (1:500; Santa Cruz Biotechnology, sc-8404, Dallas, TX, USA); anti-p-NF-κB (1:500; Santa Cruz Biotechnology sc-166748, Dallas, TX, USA) and anti-IKKβ (1:500; Cell Signaling, catalog#2684; Massachusetts, USA). Antibody dilutions were made in PBS/5% *w*/*v* non-fat dried milk/0.1% Tween-20 (PMT) and membranes incubated overnight at 4 °C. Membranes were then incubated with secondary antibody (1:2000, Jackson ImmunoResearch, West Grove, PA, USA) for 1 h at room temperature. To ascertain those blots were loaded with equal amounts of protein lysate, they were also incubated with β-actin antibody (for cytosolic fraction 1:500; Santa Cruz Biotechnology, Dallas, TX, USA; sc-8432) or lamin A/C (for nuclear fraction 1:500, Santa Cruz Biotechnology, Dallas, TX, USA; sc-376248). Signals were detected with enhanced chemiluminescence (ECL) detection system reagent according to the manufacturer’s instructions (Thermo Fisher, Waltham, MA, USA). The relative expression of the protein bands was quantified by densitometry with BIORAD ChemiDocTMXRS + software. The densitometric values are normalized with Lamin A/C and β-actin and expressed as % of the control.

#### 4.2.6. Immunohistochemical Localization for E-Cadherin and N-Cadherin

Immunohistochemical localization was performed, as previously described [59]. Slides were incubated overnight (O/N) using the following primary antibodies: anti-E-cadherin (Santa Cruz Biotechnology, Dallas, TX, USA; 1:100 in PBS, *v*/*v*; sc-8426) and anti-N-cadherin (1:100; Santa Cruz Biotechnology, Dallas, TX, USA; sc-8424). At the end of the incubation with the primary antibodies, the sections were washed with PBS and incubated with a secondary antibody (Santa Cruz Biotechnology, Dallas, TX, USA) for 1 h at room temperature. The reaction was revealed by a chromogenic substrate (brown DAB), and counterstaining with nuclear fast-red. Immunohistochemical images were obtained and observed using a Zeiss microscope with Axio Vision software. The percentage area of immunoreactivity (brown staining, determined by the number of positive cells) is expressed as % of the total tissue area (red staining) of five random fields with objective lens at 20 × (50 μm of the bar scale) and 40× (20 μm of the bar scale); the analysis was performed using ImageJ.

#### 4.2.7. Enzyme-Linked Immunosorbent Assay (ELISA) for NIK, IL-1β, TNFα and SOD

The levels of NIK, IL-1β, TNF-α and SOD were measured by ELISA kit according to the manufacturer’s instructions (Mouse NIK ELISA kit cat. No. E060321; Biobool; Mouse IL-1β ELISA kit cat. ab197742; Abcam; UK; Mouse TNFα ELISA kit cat. No. MBS825075 MyBiosource; San Diego, CA, USA; Mouse Superoxide Dismutase (SOD) ELISA kit cat. No MBS034842 MyBiosource; San Diego, CA, USA). Samples were thawed on ice, homogenized in 300 μL lysis buffer and centrifuged at 14,000× g for 10 min at 4 °C; then, supernatants were collected and stored at −20 °C.

#### 4.2.8. Malondialdehyde (MDA) Assay

Malondialdehyde (MDA) content was evaluated in the homogenate supernatant of the tumor samples by colorimetric reaction with thiobarbituric acid (TBA). The MDA content was measured at a wavelength of 532 nm, as previously described [61,62].

#### 4.2.9. Reactive Oxygen Species (ROS) Assay

ROS evaluation was performed in tumor samples using Reactive Oxygen Species (ROS) Assay Kit (Cat. No.: CB-P048-K, Creative Biolabs) according to manufacturer’s instructions.

#### 4.2.10. Statistical Analysis

All values are expressed as mean ± standard error of the mean (SEM) of “n” observations. Each analysis was performed three times with three samples replicates for each one. The results were analyzed by one-way analysis of variance (ANOVA) followed by a Bonferroni post hoc test for multiple comparisons. A *p*-value of less than 0.05 was considered significant.

### 4.3. Ex Vivo Studies

#### 4.3.1. Patient-Derived Glioblastoma Cell Culture

Primary tumor GB cells from patients were acquired according to protocol approved by the Regional Ethical Board at the University of Messina. All subjects gave their informed consent for inclusion before they participated in the study. The study was performed in accordance with the Declaration of Helsinki, and the protocol was approved by the Ethics Committee of AOU “G. Martino,” Hospital of Messina (No. 47/19 of 05/02/2019). Tumor samples were processed aseptically, and primary cell cultures were initiated using DMEM (Catalog No. D5030; Sigma-Aldrich) with 15% heat-inactivated fetal calf serum (FCS) (Catalog No. 12103C; Sigma-Aldrich), 2 mM GlutaMAX-I (Catalog No. 35050061; ThermoFisher Scientific), 1% insulin-transferrin-selenium-X supplement (Catalog No. 41400045; ThermoFisher Scientific), and 1% penicillin-streptomycin mixture (Catalog No. 15640055; Invitrogen, Carlsbad, CA, USA). Cells were used within 7 days of plating or established as primary cell lines, as previously described [63].

##### Experimental Groups

1. Control group: primary GB cells obtained from patients were treated with culture medium.
2. Vehicle group: primary GB cells obtained from patients were treated with 0.001% of DMSO dissolved in culture medium.
3. GSK343 1 μM: GB cells from patients were treated with GSK343 at concentration of 1 μM dissolved in culture medium with 0.001% of DMSO.
4. GSK343 10 μM: GB cells from patients were treated with GSK343 at concentration of 10 μM dissolved in culture medium with 0.001% of DMSO.
5. GSK343 25 μM: GB cells from patients were treated with GSK343 at concentration of 25 μM dissolved in culture medium with 0.001% of DMSO.

#### 4.3.2. Cell Viability Assay

Primary GB cells culture obtained from patients were treated with GSK343 at the concentrations of 1, 10 and 25 μM dissolved in culture medium with 0.001% of DMSO for 24 h. After 24 h, primary GB cells were incubated at 37 °C with MTT (0.2 mg/mL; M5655; Sigma-Aldrich) for 1 h. The medium was removed by aspiration, and the cells were lysed with 100 μL of DMSO (sc-358801; Santa Cruz Biotechnology). The extent of reduction of MTT to formazan was quantified by measurement of optical density at 550 nm (OD550) with a microplate reader as described [63].

#### 4.3.3. Enzyme-Linked Immunosorbent Assay (ELISA) for CXCL9, CXCL10 and CXCL11

The levels of CXCL9, CXCL10 and CXCL11 were measured in primary GB cells supernatant by ELISA assay according to manufacturer instructions (Human CXCL9 ELISA kit, Catalog No. EHCXCL9, Invitrogen; Human CXCL10 ELISA kit, Catalog N. CHC2363; Invitrogen; Human CXCL11 ELISA kit, Catalog No. LS-F23762, LSBio).

### 4.4. Materials

GSK343 and other chemical reagents are purchased by Sigma-Aldrich (Milan, Italy). All stock solutions were prepared in non-pyrogenic saline (0.9% NaCl; Baxter, Liverpool, UK).

### 4.5. Statistical Analysis

All values are expressed as mean ± standard error of the mean (SEM) of “n” observations. Each analysis was performed three times with three samples replicates for each one. The results were analyzed by one-way analysis of variance (ANOVA) followed by a Bonferroni post hoc test for multiple comparisons. A *p*-value of less than 0.05 was considered significant.

## 5. Conclusions

In conclusion, our data demonstrated, for the first time, that GSK343 treatment was able to modulate canonical and non-canonical NF-κB/IκBα pathway activation in GB. Moreover, in the field of cancer research, our preliminary data obtained about the capacity of GSK343 to modulate OS and immune response in GB appears very interesting. Therefore, based on the obtained results, GSK343, thanks to its numerous abilities, could represent a possible therapeutic strategy to contrast or reduce GB growth, which has a extremely high mortality due to its resistance to currently used therapies. However, considering the limitations of preclinical models, further studies are needed for a fuller understanding of the mechanism of action of GSK343 in GB pathology.

## Data Availability

The data presented in this study are available on request from the corresponding author.

## Supplementary Materials

The following supporting information can be downloaded at: https://www.mdpi.com/article/10.3390/ijms232213915/s1.
