# Supplementary figures and images for: GSK343, an Inhibitor of Enhancer of Zeste Homolog 2, Reduces Glioblastoma Progression through Inflammatory Process Modulation: Focus on Canonical and Non-Canonical NF-κB/IκBα Pathways
Source: Int J Mol Sci. 2022 Nov 11;23(22):13915. doi: 10.3390/ijms232213915 (PMC9694970; doi:10.3390/ijms232213915)

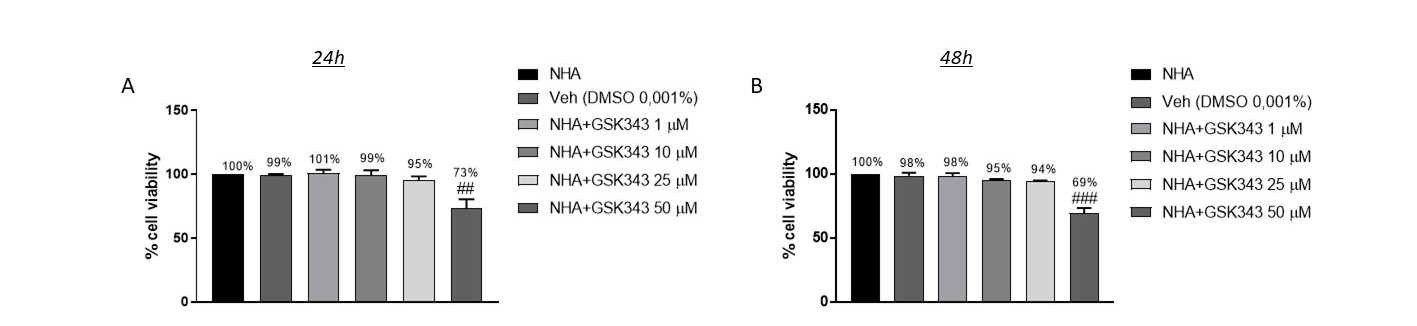

Supplement: Supplementary file 1 [file ijms-23-13915-s001.zip › Supplementary Figure 1.jpg]

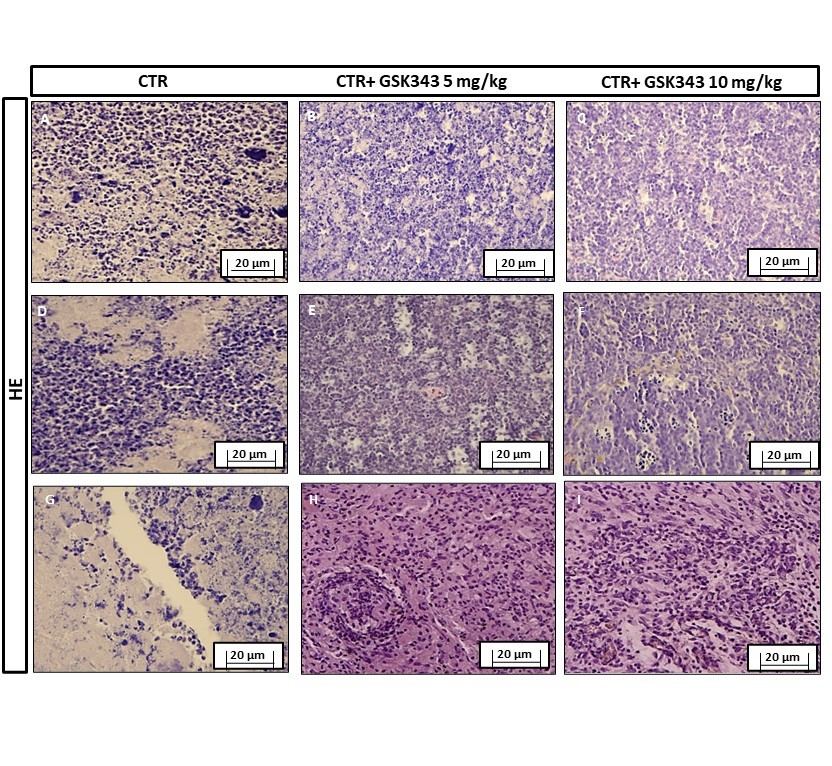

Supplement: Supplementary file 1 [file ijms-23-13915-s001.zip › Supplementary figure 2.jpg]
